# Fibrolipoma of the Buccal Space in a 47-Year-Old Male: A Case Report

**DOI:** 10.3390/reports9010034

**Published:** 2026-01-24

**Authors:** Athanasios Vlachodimitropoulos, Spyridon Lygeros, Michail Athanasopoulos, Dimitra Koumoundourou, Georgios Batsaouras

**Affiliations:** 1 Department of Otolaryngology—Head and Neck Surgery, General University Hospital of Patras, 26504 Patras, Greece; 2 Department of Pathology and Cytopathology, General University Hospital of Patras, 26504 Patras, Greece

**Keywords:** fibrolipoma, buccal fat pad, buccal space tumor, oral lipoma, intraoral excision

## Abstract

**Background and Clinical Significance:** Fibrolipoma is an uncommon histological variant of lipoma characterized by mature adipose tissue with a significant fibrous component. Intraoral lipomas are rare (only about 1–4% of all lipomas) and lipomas arising in the buccal fat pad (buccal space) are particularly uncommon. **Case Presentation:** A 47-year-old male presented with a painless, slowly enlarging swelling in the left cheek region. Physical examination revealed a soft, non-tender mass in the buccal space, causing mild bulging of the cheek. Contrast-enhanced computed tomography and magnetic resonance imaging demonstrated a well-circumscribed lesion within the left buccal fat pad suggestive of a lipoma. The tumor was excised entirely via an intraoral approach under general anesthesia. Histopathological examination showed lobules of mature adipocytes interspersed with dense fibrous connective septa consistent with a diagnosis of a fibrolipoma. The postoperative course was uneventful. **Conclusions:** This case highlights that fibrolipoma, while rare in the maxillofacial region, should be included in the differential diagnosis of buccal space tumors. Imaging studies can aid in identifying the fatty nature and extent of such lesions, but definitive diagnosis relies on histopathology. The buccal fat pad’s anatomy allows an intraoral surgical approach in appropriate cases, providing direct access and excellent cosmetic outcomes. Complete excision is curative in benign fibrolipomas, and careful surgical technique prevents injury to adjacent structures.

## 1. Introduction and Clinical Significance

Lipomas are the most common benign mesenchymal tumors of adipose tissue [1]; however, they only infrequently involve the oral cavity (approximately 1–4% of all lipomas) [2]. Oral lipomas typically present in adults, often as slow-growing, painless submucosal masses. The buccal mucosa is reported as the most favored intraoral location for these tumors [3,4]. Fibrolipoma (FLP) is a histological variant of lipoma defined by a substantial fibrous component within the fatty tumor, and it is characterized as an uncommon variant by the World Health Organization [5]. FLPs account for only about 1.6% of all lipomas in the head and neck region, making them a rare clinicopathologic entity [5]. Clinically, a FLP usually presents as a well-circumscribed, rubbery or soft mass and is often indistinguishable from a conventional lipoma except by histology [6]. While benign, FLPs have been noted to sometimes exhibit slightly higher cellular proliferation (elevated Ki-67 index) compared to ordinary lipomas [4]. This raised proliferative potential has led some authors to recommend close follow-up, as FLP might recur more frequently than other lipoma subtypes.

Lipomas arising in the buccal space are particularly uncommon [7]. The buccal space is a superficial facial space predominantly occupied by the buccal fat pad (BFP) [8]. A search of historical literature by de Wijn et al. (2009) found only 27 cases of lipomas originating in the BFP reported between 1848 and 2002 [7] and there have been limited case reports since then, highlighting the rarity of BFP lipomas (we identified 8 cases in total [9,10,11,12,13,14,15,16] searching Pubmed using the query “buccal AND *lipoma*”). Because of this rarity and some atypical presentations, BFP lipomas may be under-recognized clinically. We report a case of a FLP arising in the BFP of a 47-year-old male. This case is notable due to its location within the buccal space and the successful use of an intraoral approach for tumor excision. We discuss the tumor’s characteristics, the relevant anatomy and differential diagnosis of buccal space masses, and the considerations that guided our surgical management.

## 2. Case Presentation

A 47-year-old male was referred to our clinic with a complaint of a persistent swelling in his left cheek. The patient first noticed a small lump inside his cheek approximately 2 years prior, which had gradually increased in size. There was no history of acute trauma to the face. He denied pain, numbness, or drainage. The main concern was a visible bulge on the left cheek that had become cosmetically bothersome.

On extraoral inspection, a subtle fullness was noted over the left lower buccal region compared to the right side. The overlying skin was normal with no discoloration and a soft, moveable, well-circumscribed mass could be palpated. Intraoral examination revealed a subtle elevation of the left lower buccal mucosa in the lower buccal sulcus. Palpation revealed a pliable, well-defined mass approximately 3–4 cm in diameter in the buccal vestibule, extending towards the cheek. The lesion was non-tender, and the mucosa overlying it was intact with normal coloration. No ulceration or distinct encapsulated structure was visible in the mouth. There were no signs of fixity to the skin or mucosa, and no regional lymphadenopathy was present. The clinical impression was a benign soft tissue tumor of the buccal space.

**Figure 1:**
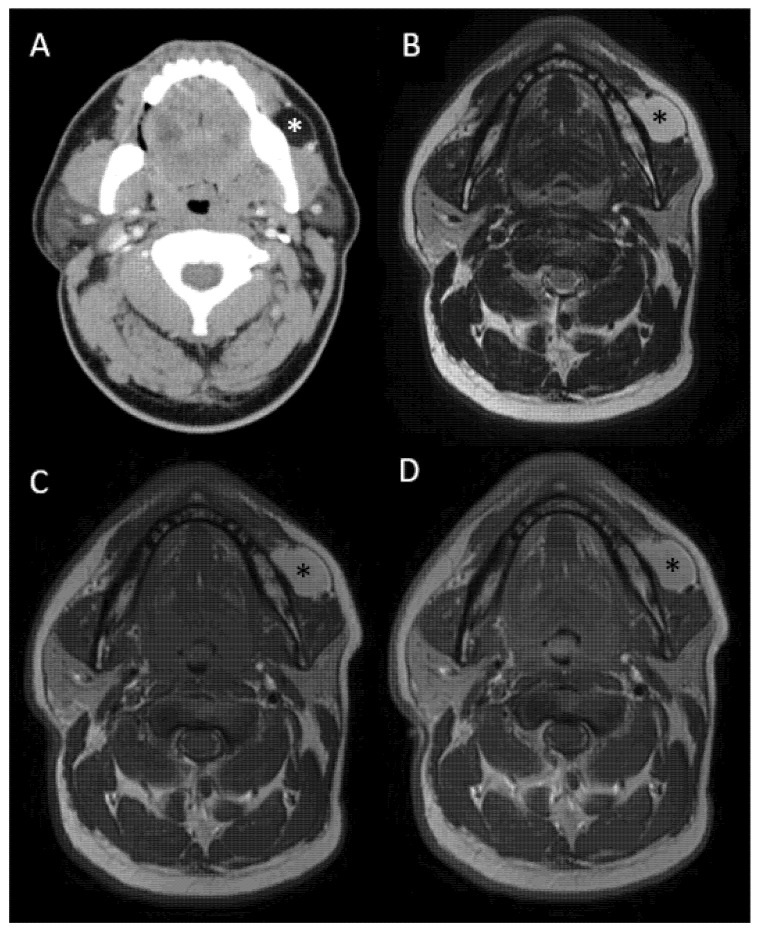
(**A**) Post contrast CT scan showing a homogeneous, low-attenuation mass (2.2 × 4.1 × 2.0 cm) in the left buccal space (asterisk). (**B**) On T1- and (**C**) T2-weighted MRI sequences, the mass had a high signal (asterisk) and (**D**) did not enhance after gadolinium administration on the T1-weighted, fat suppressed, post-contrast sequence (asterisk).

To characterize the lesion, a contrast-enhanced computed tomography (CT) scan and a Magnetic Resonance Imaging (MRI) scan were conducted. The CT scan showed a homogeneous, low-attenuation mass (2.2 × 4.1 × 2.0 cm) in the left buccal space, with Hounsfield units (HUs) consistent with adipose tissue (−80 HUs) (Figure 1). Following intravenous contrast administration, no measurable enhancement was observed, defined as an absence of interval increase in attenuation greater than 10 HUs between pre- and post-contrast images. The lesion was well-demarcated and caused a mild outward contour bulge of the cheek, without bone erosion. MRI provided further detail: on T1-weighted images, the mass had a high signal similar to normal fat and it showed complete signal loss on fat-suppressed sequences, confirming its fatty nature. On T2-weighted images, the lesion also exhibited a high signal and there was no enhancement or invasive feature on the T1-weighted fat suppressed sequence after gadolinium administration. The mass appeared to be confined to the BFP compartment, situated lateral to the buccinator muscle and anterior to the masseter, with a clear plane separating it from the surrounding musculature (Figure 1). The parotid gland and Stensen’s duct were unremarkable on imaging and were displaced superiorly by the lesion. No satellite lesions or suspicious lymph nodes were noted. These radiologic findings were highly suggestive of a benign lipomatous tumor originating in the BFP.

**Figure 2:**
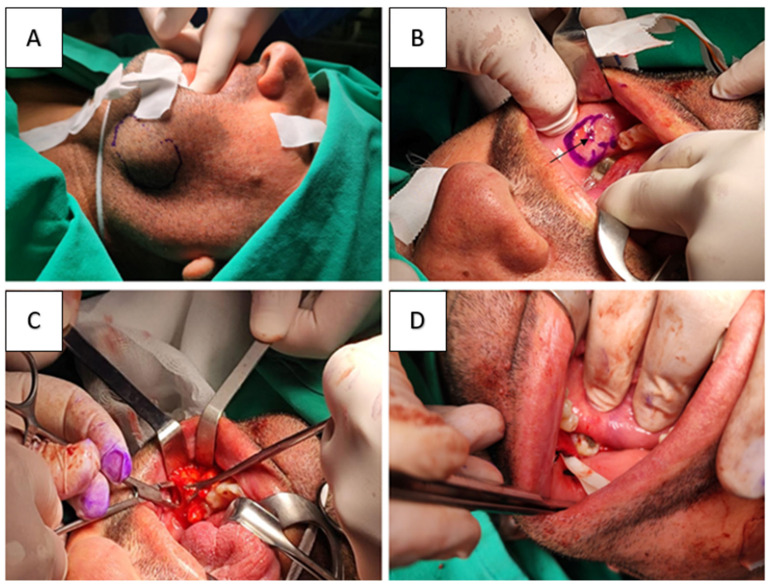
(**A**) Extraoral inspection shows a mass in the left lower cheek. (**B**) Identification of the parotid duct papilla (arrow). (**C**) A multilobulated fatty mass was identified after dissection through the mucosa and the buccinator muscle. (**D**) Wound closure and drainage placement.

Given the tumor’s benign characteristics and well-defined nature, surgical excision was planned both for definitive diagnosis and to relieve the patient’s symptoms. An intraoral approach was selected to avoid external scarring. Under general anesthesia with endotracheal intubation, the patient was placed in a supine position. A mouth gag and retractors were used to provide access to the left lower buccal sulcus. After infiltration with a local anesthetic solution containing epinephrine for hemostasis, a vertical incision (~4 cm) was made in the left lower buccal mucosa, in the vestibule below and anterior to the parotid duct papilla. After careful blunt dissection through the buccinator muscle, the BFP was exposed and the lipomatous lesion was identified within it (Figure 2). Although the tumor was well-encapsulated radiologically, intraoperatively it proved difficult to deliver as a single mass due to its lobulated architecture and its intimate attachment to surrounding buccal fat. Therefore, the lesion was removed by fragmented excision of individual lobules while preserving the parotid duct and buccal branches of the facial nerve. All grossly identifiable lipomatous tissue was removed, achieving complete macroscopic excision. Hemostasis was achieved and the mucosal incision was closed with resorbable sutures.

**Figure 3:**
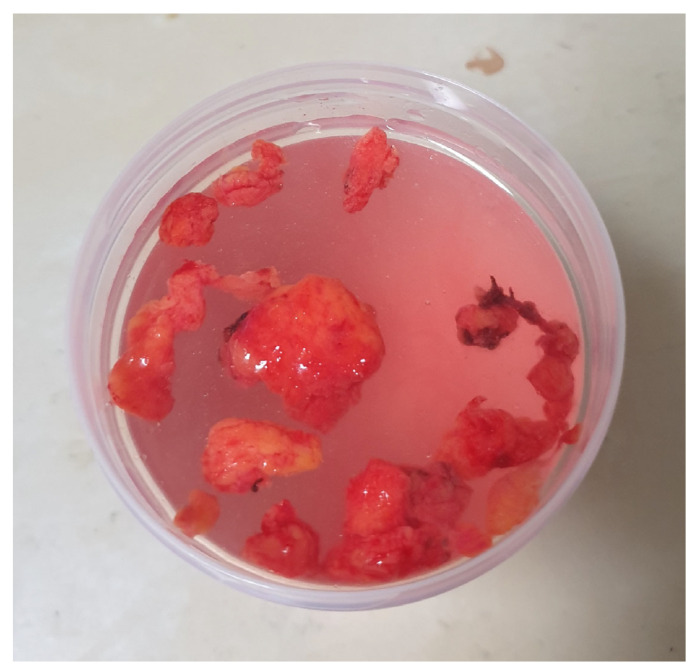
Gross specimen consisting of multiple lobulated fragments of adipose tissue removed by fragmented excision from the left buccal fat pad.

On gross examination, the resected specimen consisted of multiple lobulated, yellow-tan adipose tissue fragments obtained by fragmented removal of individual lobules. The cumulative volume of the excised tissue was approximately 10.5 cm^3^ (Figure 3). Some were encapsulated and had fibrous septations visible on the cut surface.

**Figure 4:**
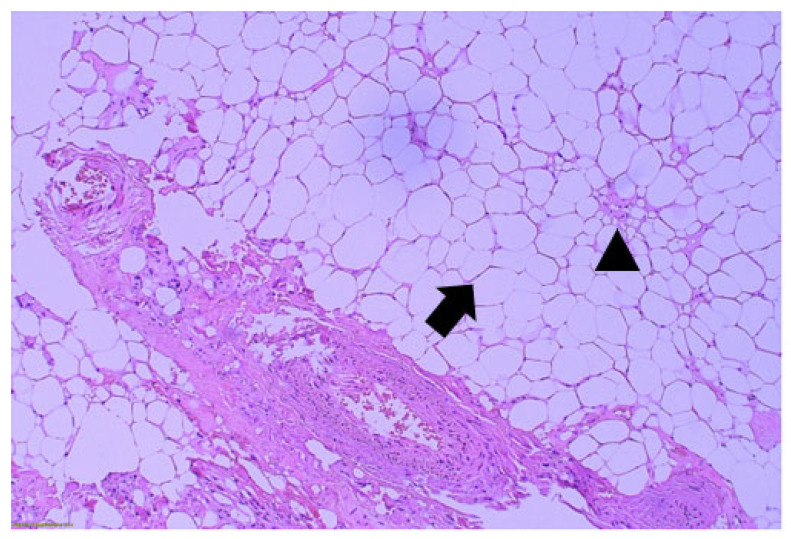
A fairly homogeneous and well-circumscribed neoplasm was recognized with proliferation of mature adipocytes (arrow) and presence of paucicellular fibrous septa (arrowhead). The adipocytes were roughly the same size, with small bland nuclei compressed at the periphery of the cell and rare nuclear vacuolization (Lockhern change). Neither atypia nor mitoses were observed (original magnification ×200).

Histopathologic examination demonstrated a well-circumscribed lesion composed of mature adipocytes interspersed with dense fibrous connective tissue, consistent with fibrolipoma. The adipocytes were uniform in size and shape without cytologic atypia, pleomorphism, hyperchromasia, or increased mitotic activity. No lipoblasts were identified on routine hematoxylin and eosin staining. The fibrous component consisted of collagenous bands separating lobules of mature fat without evidence of infiltrative growth (Figure 4). These findings fulfill the established histologic criteria for fibrolipoma and effectively exclude atypical lipomatous tumor or well-differentiated liposarcoma. Although the lesion was well-circumscribed histologically, surgical margin assessment was not feasible due to fragmented removal.

The patient’s postoperative recovery was smooth. There was minimal cheek swelling and no signs of hematoma or sialocele. The patient was discharged the next day on oral antibiotics and analgesics. At the 1-week follow-up, the oral incision had healed well with no signs of infection. By one month, the patient reported normal cheek sensation and function, and was pleased with the restoration of facial symmetry. We observed no facial nerve deficit and an intact Stensen’s duct. At a 6-month follow-up visit, there was no evidence of recurrence or residual mass. The surgical site appeared fully healed, and the patient remained asymptomatic. Periodic follow-up was advised given that fibrolipomas, while rare to recur, have been noted to warrant monitoring [4].

## 3. Discussion

Intraoral lipomas are uncommon benign tumors, and FLP is recognized as a distinct variant with unique histologic features. In FLPs, the presence of a substantial fibrous stroma distinguishes them from conventional lipomas [17]. This fibrous content can confer a firmer consistency and can even alter imaging characteristics (for example, FLPs may appear with areas of lower T2 signal on MRI due to the collagen, unlike pure fat lesions) [18]. Clinically, however, FLPs generally present like other lipomas: a painless, slow-growing, well-circumscribed mass that is mobile and non-tender. Patients often seek treatment for aesthetic reasons or if the mass causes functional interference (difficulty chewing, speaking) [19]. In our patient, the FLP was asymptomatic aside from the cheek bulge, consistent with the literature that these tumors usually only become noticeable when they reach a size causing cosmetic or functional concern. The etiopathogenesis of lipomas, including FLPs, is not definitively established. Proposed factors include trauma, chronic irritation, hormonal influences, and genetic predispositions [20].

The buccal space is a distinct superficial facial compartment with well-defined fascial boundaries. Medially, it is bounded by the buccinator muscle and its overlying fascia, representing an extension of the buccopharyngeal fascia. Laterally, the space is limited by the superficial musculoaponeurotic system (SMAS), including the platysma inferiorly and the midfacial aponeurotic layer superiorly. Posteriorly, the buccal space is separated from the masticator space by the anterior lamina of the parotido-masseteric fascia, which forms the facial vein canal; notably, the facial vein courses within this posterior boundary rather than within the space itself. Anteriorly, the boundary is formed by the modiolus, where multiple muscles of facial expression converge. Superiorly, a transverse fascial septum extends from the undersurface of the zygomaticus major muscle to the alveolar process of the maxilla, forming a consistent superior limit. Inferiorly, the boundary is multipartite, consisting anteriorly of the platysma’s firm bony adhesion to the mandible and the mandibular ligament, while, posteriorly, the facial vessels cross the mandible in an area lacking strong platysmal attachment [21]. This space is largely occupied by the BFP, also known as Bichat’s fat pad [22]. The BFP is an encapsulated collection of adipose tissue with a central body and several extensions that spread into adjacent regions of the face. It serves both functional and aesthetic roles: it facilitates smooth movement of facial muscles (by filling gaps and reducing friction during mastication) and provides soft tissue fullness to the cheek contour [7]. Several important structures are intimately associated with the buccal space including the facial artery and vein, the parotid duct, and buccal branches of the facial nerve. The parotid (Stensen’s) duct travels through the BFP, running laterally along the BFP before it pierces the buccinator muscle to enter the oral cavity opposite the upper second molar. The buccal branches of the facial nerve also course along the superficial surface of the BFP (within or deep to the superficial fascia) to innervate the muscles of facial expression in the cheek region [23]. These anatomical relationships are critical when planning surgical approaches to buccal space tumors to avoid injuring the parotid duct or facial nerve fibers.

A mass in the buccal space or cheek can arise from a variety of tissue origins, so a broad differential diagnosis must be considered [7]. Lipomatous lesions are one category, which includes simple lipoma and its variants (FLP, angiolipoma, spindle-cell lipoma, etc.) [22]. Although exceedingly rare in the buccal area, a well-differentiated liposarcoma can mimic a benign lipoma [24]. Clues to a malignant fatty tumor include a more infiltrative growth pattern, lack of a neat lobular architecture, presence of lipoblast cells, rapid enlargement, pain and ulceration [6]. In the BFP specifically, one must also distinguish a true tumor from a herniation of the BFP as well as a traumatic pseudolipoma. BFP herniation (pseudoherniation) can present as a cheek mass, often in older adults, but it represents prolapse of normal fat rather than a neoplasm due to loss of ligamentous support [25]. Traumatic pseudolipoma is an intraoral herniation of the BFP, often in children, related to trauma that requires the piercing of both the buccinator muscle and oral mucosa [8].

Other important considerations in the differential diagnosis of a buccal space swelling include a range of soft tissue lesions (fibroma, neurofibroma, neuroma, sebaceous adenoma) [26,27,28], salivary gland benign and malignant neoplasms (pleomorphic adenoma, oncocytoma, basal cell adenoma, adenoid cystic carcinoma, acinic cell carcinoma, mucoepidermoid carcinoma, carcinoma ex pleomorphic adenoma, salivary duct carcinoma, adenocarcinoma) [22], vascular lesions (hemangioma, lymphangioma) [28], cysts and developmental lesions (dermoid and epidermoid cyst, hamartoma) [29,30], and inflammatory, lymphoid and metastatic lesions [22].

Complete surgical excision is the treatment of choice for lipomas and their variants in the oral and maxillofacial region [31]. Given the location of our patient’s tumor in the buccal space, we had to decide between an intraoral and an external (transcutaneous) approach. An intraoral (transoral) approach was utilized, which is supported by the literature as an effective and common route for buccal space tumors [22]. Approaching through the mouth offers several advantages: it provides direct access to the BFP from inside the cheek, avoids any visible external scar, and generally involves less extensive dissection through facial tissues [22]. In fact, a review of reported buccal space lipoma cases shows that the majority were managed via intraoral excision, emphasizing its feasibility and safety [7].

However, it is crucial to consider the limitations and precautions of the intraoral approach. Visualization of certain structures is limited from inside the mouth; specifically, the surgeon cannot directly see the facial nerve branches or the proximal course of Stensen’s duct during the approach [22]. As a result, careful knowledge of their anatomy is required to avoid injury. In our procedure, we took care to locate the parotid duct papilla and make our incision well below it and use blunt dissection to avoid traction on any nerve fibers. Fortunately, the BFP lies deep to (medial to) the plane of the facial nerve and duct in most cases, meaning a properly executed intraoral dissection can remove an encapsulated BFP lesion without encountering those structures. Still, one must be vigilant: a report by Hwang et al. noted that in roughly 26% of individuals, portions of the parotid duct or buccal nerve may run unusually medial to the BFP [32]. This anatomical variation underscores why gentle technique and, in some cases, preoperative imaging (to map the duct) are important.

For very large buccal space tumors, tumors with diffuse extensions, or cases where a malignancy is suspected, an external approach may be recommended [22]. Options include a modified preauricular (extended parotidectomy) incision or a facelift (rhytidectomy) approach, which provide wider exposure of the buccal space. These approaches allow the surgeon to clearly visualize and protect the facial nerve and parotid duct and to access deeper extensions of the BFP if needed. The trade-off is a larger incision with potential visible scarring and a more invasive dissection [22]. Our patient’s FLP was well-encapsulated and localized, making it ideal for transoral excision. We avoided any external incisions and encountered no complications. The operation time was relatively short, and the patient’s recovery was quick.

Recurrence of lipomas in the oral cavity is rare, and when it occurs, incomplete removal is often the cause [6]. In FLPs, because of the fibrous component, some have a slightly more infiltrative growth pattern (or can have multiple lobules in different extensions of the fat pad) [7]. If any portions are left behind, the lesion could potentially regrow. Indeed, other authors have emphasized the importance of understanding the multilobulated anatomy of the BFP so that all tumor extensions are resected [33]. While ordinary oral lipomas rarely recur, FLPs have been reported to exhibit a slightly “greater proliferative activity” than other variants. Thus, some authors recommend diligent follow-up after removal of an FLP [4]. In the present case, the fibrolipoma was removed by fragmented excision due to its multilobulated architecture and continuity with the buccal fat pad. This approach, while appropriate for a benign lesion, precludes formal histologic margin assessment. Therefore, completeness of excision was determined on a macroscopic and clinical basis, with absence of recurrence at follow-up serving as the primary indicator of successful treatment. Malignant transformation of a lipoma into liposarcoma in the oral region is exceedingly rare, but there are isolated reports (especially in long-standing untreated lesions) [34]. Therefore, thorough histopathological analysis is essential to confirm the diagnosis and rule out any atypical features.

Our case adds the following points of practical relevance. First, it provides a comprehensive imaging–pathology correlation, illustrating how a fibrolipoma of the buccal fat pad can demonstrate imaging characteristics indistinguishable from a simple lipoma on CT and MRI, underscoring the necessity of histopathologic confirmation. Second, this case details technical nuances of a transoral surgical approach to a multilobulated lesion within the buccal fat pad, including the need for fragmented removal of individual lobules and the implications this has for margin assessment in benign tumors.

## 4. Conclusions

FLP of the buccal space is a rare benign tumor that should be considered in the differential diagnosis of cheek masses. This case illustrates that such a lesion can be managed successfully with an intraoral surgical approach, avoiding external scars while achieving complete removal. Knowledge of the buccal space anatomy—particularly the location of the BFP and its relationship to the parotid duct and facial nerve—is critical for safe surgery. Preoperative imaging (CT/MRI) is extremely helpful to confirm the fatty nature of the tumor, delineate its extent, and rule out features suggestive of malignancy. Definitive diagnosis, however, relies on histopathological examination, which in our patient confirmed the FLP subtype. The postoperative prognosis is excellent after complete macroscopic excision, with a low recurrence potential reported for FLPs. The key lessons from this case are to maintain a broad differential for buccal space tumors (including lipomatous lesions among others) and to tailor the surgical approach to the lesion’s characteristics.

## Data Availability

The data that support the findings of this study are available upon request from the corresponding author. The data are not publicly available due to privacy or ethical restrictions.

## Abbreviations

The following abbreviations are used in this manuscript:

FLP	Fibrolipoma
BFP	Buccal Fat Pad
CT	Computed Tomography
MRI	Magnetic Resonance Imaging
HUs	Hounsfield Units
